# Characterization of m6A in mouse ovary and testis

**DOI:** 10.1002/ctm2.141

**Published:** 2020-08-12

**Authors:** Xiaofeng Sun, Jiaona Zhang, Yong Jia, Wei Shen, Hongguo Cao

**Affiliations:** ^1^ College of Life Sciences Institute of Reproductive Sciences Qingdao Agricultural University Qingdao China; ^2^ Anhui Province Key Laboratory of Local Livestock and Poultry Genetic Resource Conservation and Bio‐breeding College of Animal Science and Technology Anhui Agricultural University Hefei China

Dear Editor

To illuminate the m6A dynamic and to better optimize the development environment of sperm and oocyte in vitro, we systematically analyzed the m6A level in genital ridges, ovaries and, testes and detected the expression levels of several modification enzymes at different key stages. N6‐methyladenosine (m6A) is one of the hottest researched RNA modifications; however, the development of the roles of m6A has been long obstructed due to the lack of the effective methods for sequencing and location of m6A,^1^ as m6A reverse transcribes to a T during reverse transcription.^2^ Until 2012, m6A antibody based high‐throughput sequencing method brought an impetus to the upsurge in m6A research.^3^, ^4^ m6A is widely conserved among eukaryotic species that range from yeast, flies to mammals,^5^ suggesting the important roles in eukaryotic species. M6A is the reversible RNA modification,^6^ which can be methylated by m6A methyltransferase (METTL3, METTL14) and demethylated by m6A demethylase (FTO, AlkB). The m6A levels could be effectively decreased by either METTL3 or METTL14 knockdown.^7^ Demethylases play a role in reversing m6A. FTO erases m6A near splicing junctions, leading to exclusion of exons of alternatively spliced genes.^8^ ALKBH5 silence causes downregulation of global RNA, disruption of RNA export, and increased nascent RNA synthesis.^9^ But so far, the m6A RNA epigenetic modification during the development of sperm and oocyte has not been well studied.

The morphological and histologic features of genital ridges, ovaries, and testes were detected by HE staining and they were in accordance with those at the corresponding stages (Figures S1‐S5). At luteal phase, many luteal could be seen in the sections of mouse ovaries, meanwhile, the number of growing follicles decreased (Figure S2). Ovaries of follicular phase were obtained by PMSG treatment. After PMSG treatment, large mature follicles were prominent in the surface of the ovary, and the number of follicles had an obvious increase (Figure S3), which indicated the effective growing of follicles induced by PMSG.^10^

The dynamic status of m6A during the development of ovary and testis were tested by dot‐blot hybridization (Figure 1A‐D) and LC‐MS quantitative analysis (Figure 1E‐H). The dot‐blot results indicated that in ovaries, the level of m6A increased from 12.5 dpc to 7 dpp and reached its peak in the ovary of mature period. The difference between luteal phase and 12.5 dpc was significant (Figure 1B). Interestingly, in mature period, luteal phase was much higher than follicular stage (Figure 1B), while, in the testes of different stages, the m6A level increased from 12.5 dpc to 7dpp and reached its maximum level in the testis of adult (Figure 1D). From LC‐MS quantitative analysis, the m6A level increased with age in both female and male. At the luteal phase, the ovarian m6A reached the maximum level (Figure 1E,F). And in male, the adult testis had the highest m6A level (500 m6A per 1 000 000 A), which was significantly different from that in the male genital ridges of 12.5 dpc (Figure 1G,H). The results of immunofluorescence showed that the signal of m6A was detected in the cytoplasm and increased with age (Figures S6 and S7), and the luteal phase had the strong m6A immunofluorescence signal in the oocyte cytoplasm (Figures S6K, L).

**FIGURE 1:**
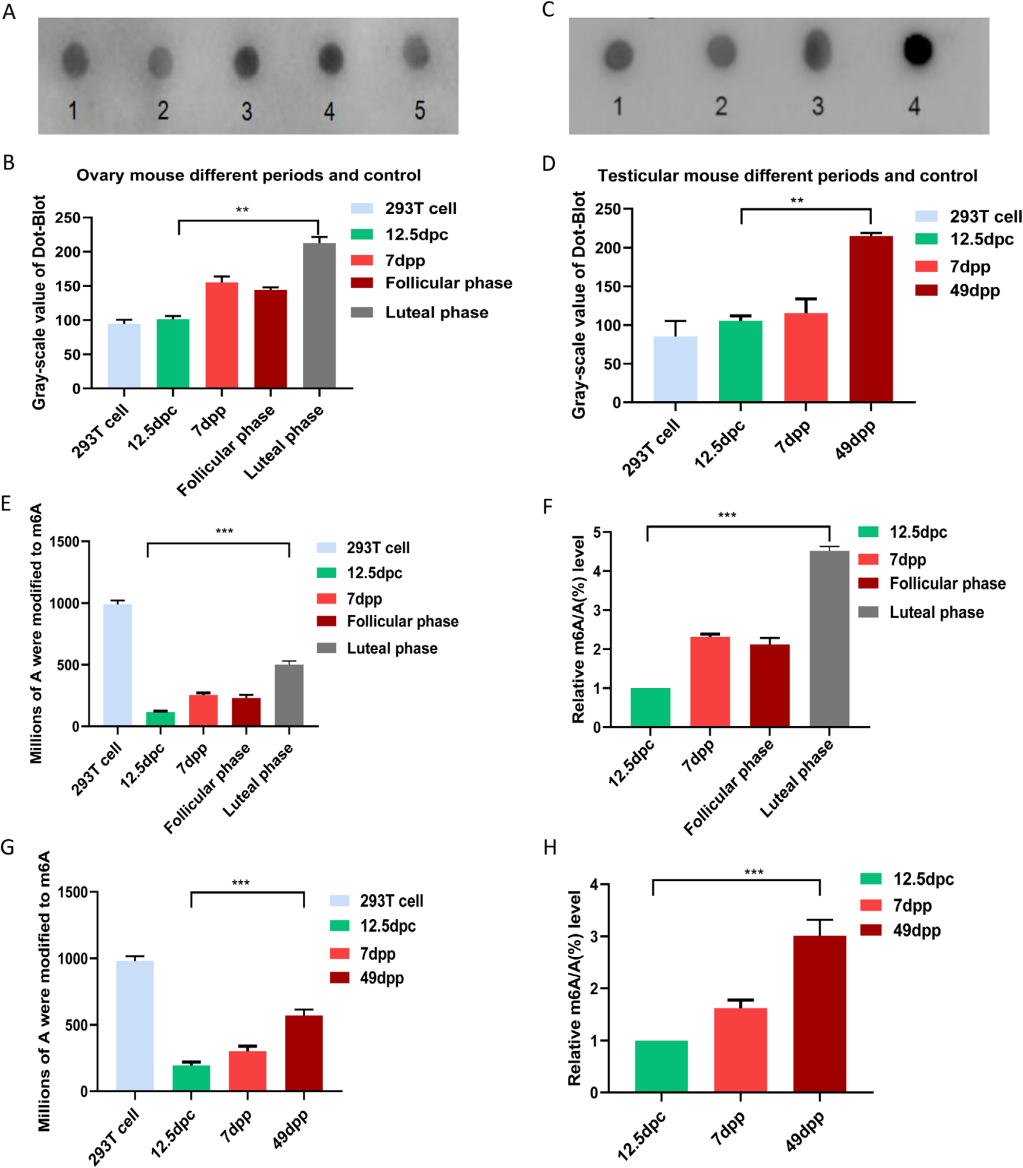
Quantitative analysis of m6A in genital ridges/ovaries/testes of different stages. A, Dot‐blot hybridization in genital ridges/ovaries of different stages. A1‐A5 were 200 ng samples of 293T cell, 12.5 dpc genital ridge, 7dpp ovary, luteal phase ovary, follicular phase ovary. (B) Gray value statistics of m6A in genital ridges/ovaries of different stages. C, Dot‐blot hybridization in genital ridges/ testes of different stages. C1‐C4 were 200 ng samples of 293T cell, 12.5 dpc male genital ridge, 7 dpp testis, and 49 dpp testis. (D) Gray value statistics of m6A in genital ridges/testes of different stages. 293T cells as positive control group. **P < .01, n = 3. E and F, LC‐MS quantitative analysis of m6A in genital ridges/ovaries of different stages. G and H, LC‐MS quantitative analysis of m6A in genital ridges/testes of different stages. 293T cells were used as positive control. E and G, The ordinate represents the number of m6A in a million A. F and H, The ordinate represents the ratio of m6A to A. ***P < .001, n = 6

Meanwhile, the expression levels of methyltransferase and demethylase for m6A were detected by RT‐PCR and western blotting. From the results, we can see that the mRNA expressions of *mettl3* and *mettl14* in the female gonads of different stages had the same trends. They both increased from 12.5 dpc to 7dpp, and the expressions reached their peaks at luteal phase. While follicular stage had the similar expression level with that of 7 dpp (Figure 2A,B). In the male gonads of different stages, the expressions of *mettl3* and *mettl14* also increased from 12.5 dpc to 7dpp and to adult. The adult stage had the highest expression of *mettl3* and *mettl14* (Figure 2C,D). However, demethylase genes *fto* and *alkbh5* showed decreased expression from 12.5 dpp to mature period (Figure 2E‐H). The expression of METTL3 and FTO at protein levels both had the similar trend with the expression of their mRNA levels (Figure 2I‐L). In female and male, methylase METTL3 increased gradually with age (Figure 2I,K). The expression of METTL3 at luteal phase was much higher than that of follicular phase in female (Figure 2I), while demethylase FTO showed the reverse results with methylase METTL3, which decreased with age. In female, the luteal phase had the lowest expression level (Figure 2J,L).

**FIGURE 2:**
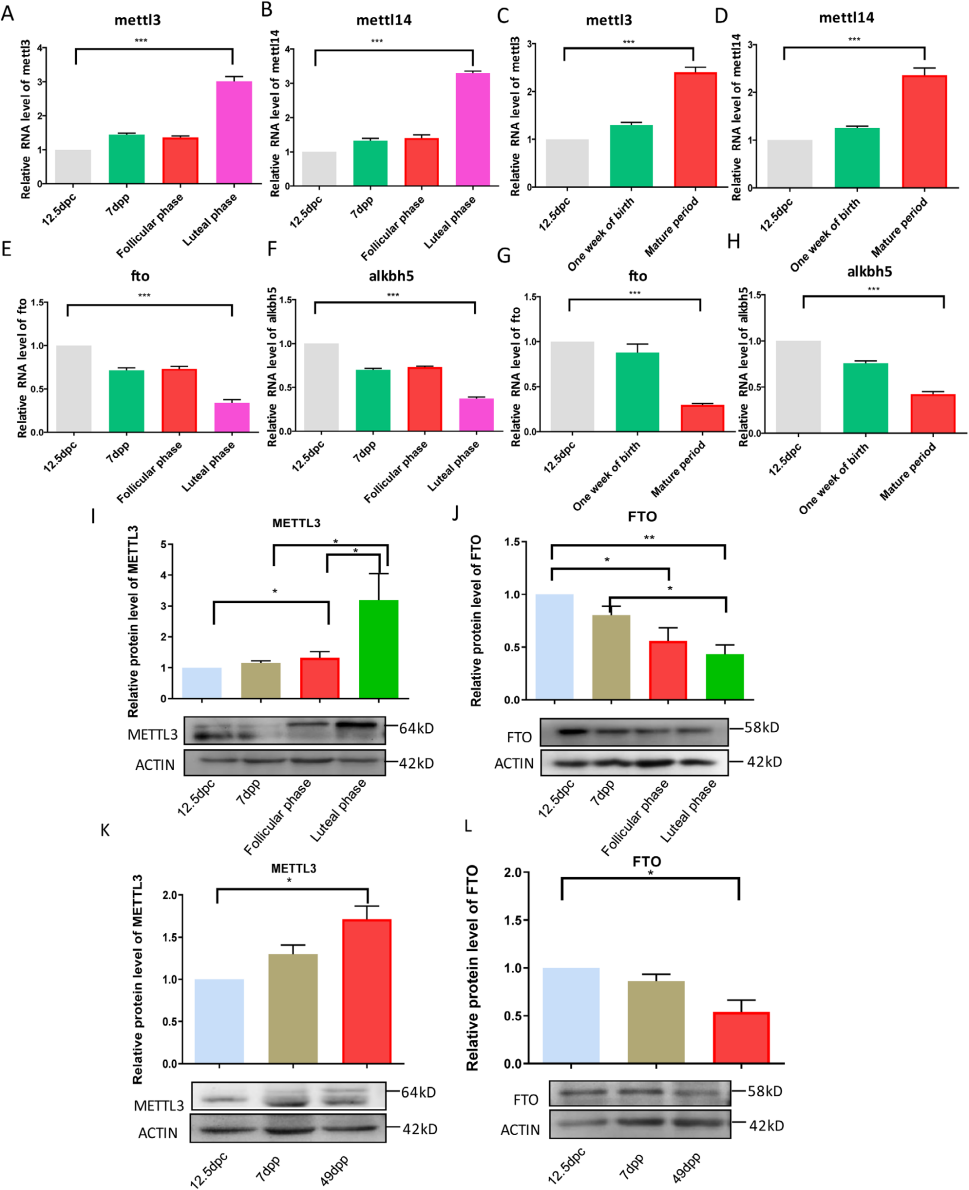
Relative expression of m6A methyltransferase genes and demethylase genes at mRNA level and protein level in genital ridges/ovaries/testes of different stages by qRT‐PCR and western blotting. A, *mettl3* mRNA in genital ridges/ovaries of different stages. B, *mettl14* mRNA in genital ridges/ovaries of different stages. C, *mettl3* mRNA in genital ridges/testes of different stages. D, *mettl14* mRNA in genital ridges/testes of different stages. E, *fto* mRNA in genital ridges/ovaries of different stages. F, *alkbh5* mRNA in genital ridges/ovaries of different stages. G, *fto* mRNA in genital ridges/testes of different stages. H, *alkbh5* mRNA in genital ridges/testes of different stages. I, METTL3 protein in ovaries of different stages. J, FTO protein in ovaries of different stages. K, METTL3 protein in testis of different stages. L, FTO protein in testis of different stages. *P < .05, **P < .01, ***P < .001, n = 3

During the development of gonads from 12.5 dpc to adult, increased methyltransferase and decreased demethylase make the m6A level increase. When methyltransferase achieved a maximal level, and demethylase decreased to its lowest point, the level of m6A reached its maximum level at the luteal stage ovary or at the adult testis. In conclusion, this study uncovered the dynamic status of m6A during the development of ovary and testis and the dynamic expression of m6A writers/eraser. All the results theoretically provide the evidence to optimize the in vitro developmental condition from m6A epigenetics.

## CONFLICT OF INTEREST

The authors declare no conflict of interest.

## AUTHOR CONTRIBUTIONS

Xiaofeng Sun analyzed the data and drafted the manuscript. Jiaona Zhang and Yong Jia developed the methodology and acquired the data. Hongguo Cao and Wei Shen designed the experiment.

## ETHICAL APPROVAL

All procedures described in the present study were reviewed and approved by the Ethical Committee of Anhui Agricultural University (agreement No. 2015–18) and Qingdao Agricultural University (agreement No. 2018‐12).

## Supporting information

Supporting Information.

## Data Availability

The data of this study are included in the article and its supported files.
