# Supplementary material for: Characterization of m6A in mouse ovary and testis
Source: Clin Transl Med. 2020 Aug 12;10(4):e141. doi: 10.1002/ctm2.141 (PMC7423183; doi:10.1002/ctm2.141)
Supplement: Supplementary file 1 — Supporting Information. [file CTM2-10-e141-s001.pdf]

Figure S1

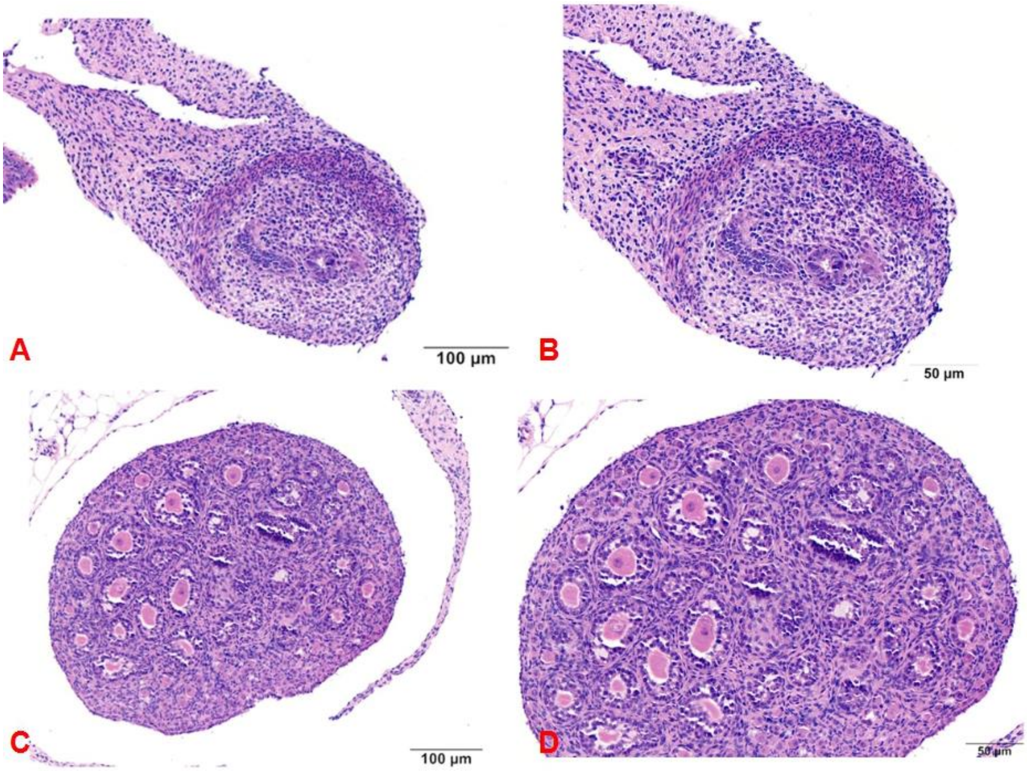

Figure S1：HE staining of female genital ridges at 12.5dpc and ovaries at 7 dpp. (A) 12.5dpc with the magnification of 100x, (B) 12.5dpc with the magnification of 200x, (C) 7dpp with the magnification of 100x, (D) 7 dpp with the magnification of 200x.

Figure S2

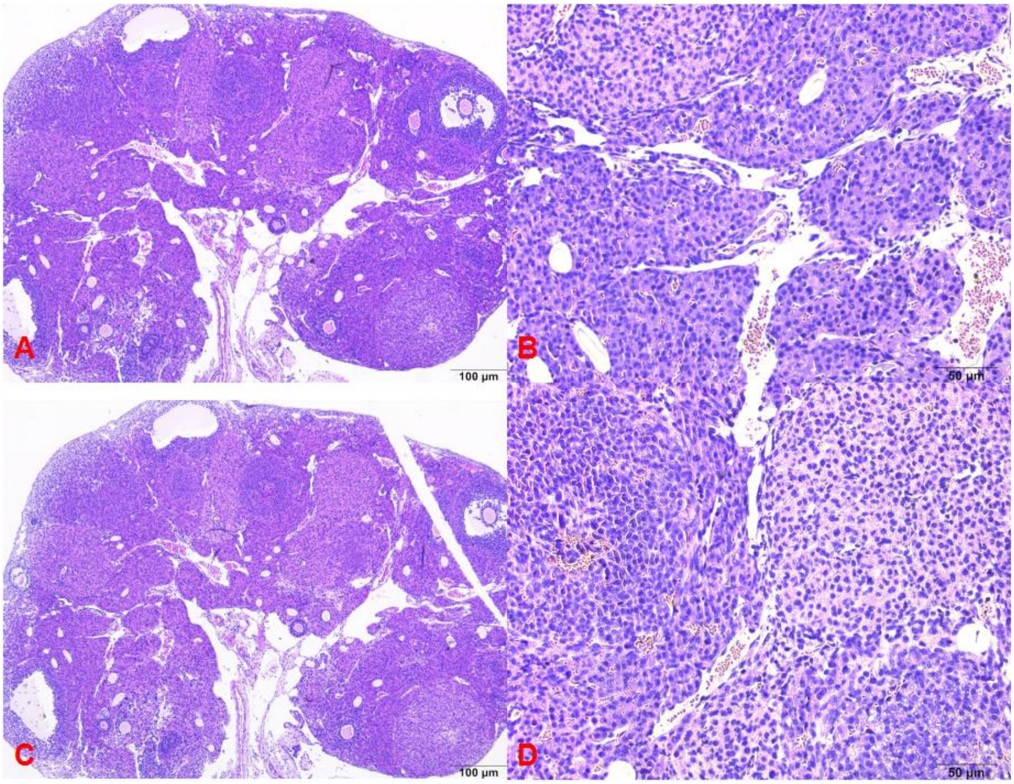

Figure S2： HE staining of ovaries at luteal phase. (A and C) with the magnification of 100x, (B and D) with the magnification of 200x.

Figure S3

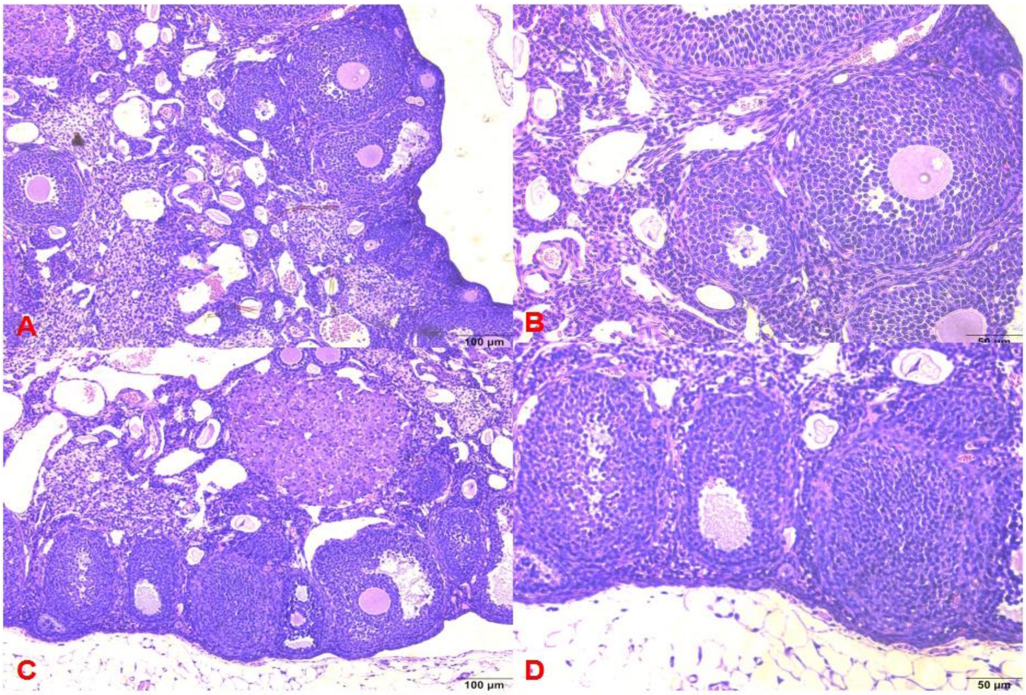

Figure S3： HE staining ovaries at follicular phase. (A and C) with the magnification of 100x, (B and D) with the magnification of 200x.

Figure S4

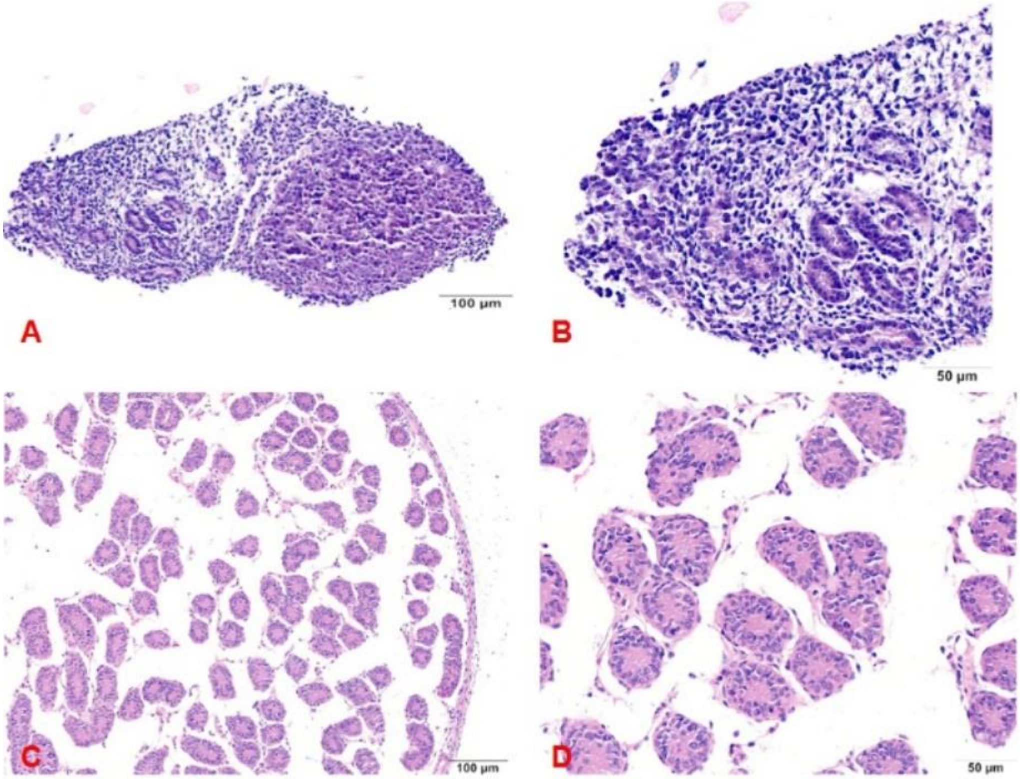

Figure S4：HE staining of male genital ridges at 12.5dpc and testes at 7 dpp. (A) 12.5dpc with the magnification of 100x, (B) 12.5dpc with the magnification of 200x, (C) 7dpp with the magnification of 100x, (D) 7 dpp with the magnification of 200x.

Figure S5

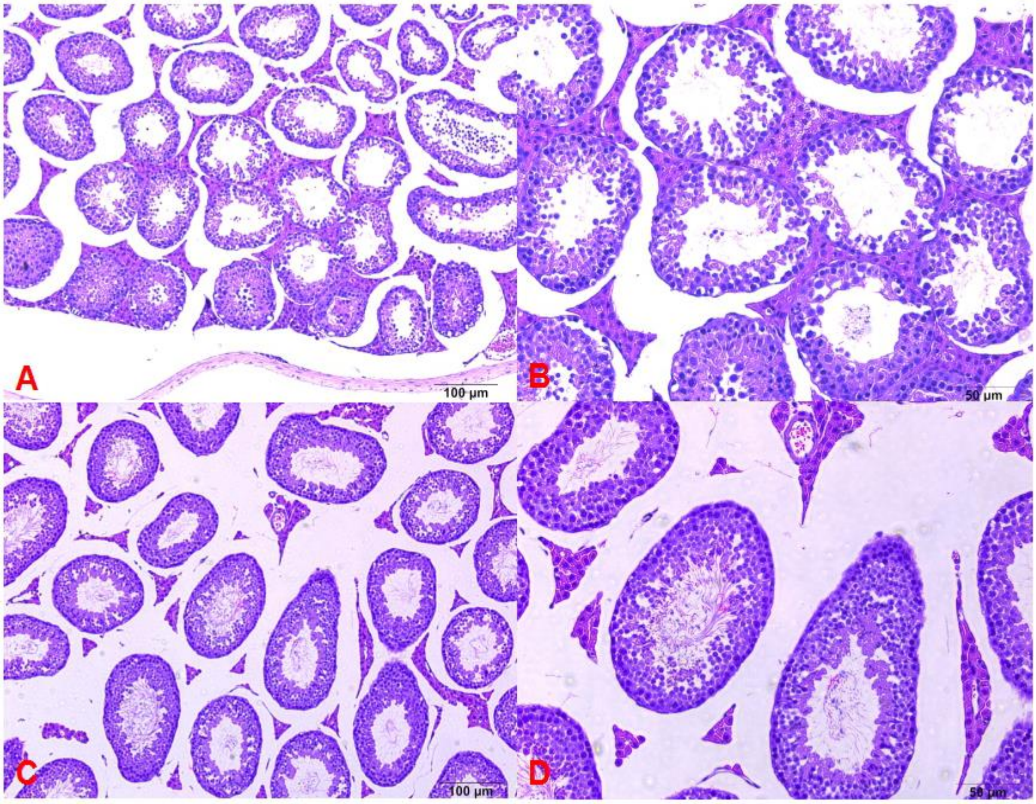

Figure S5： HE staining of adult mouse testes (49 dpp). (A and C) with the magnification of 100x, (B and D) with the magnification of 200x.

Figure S6

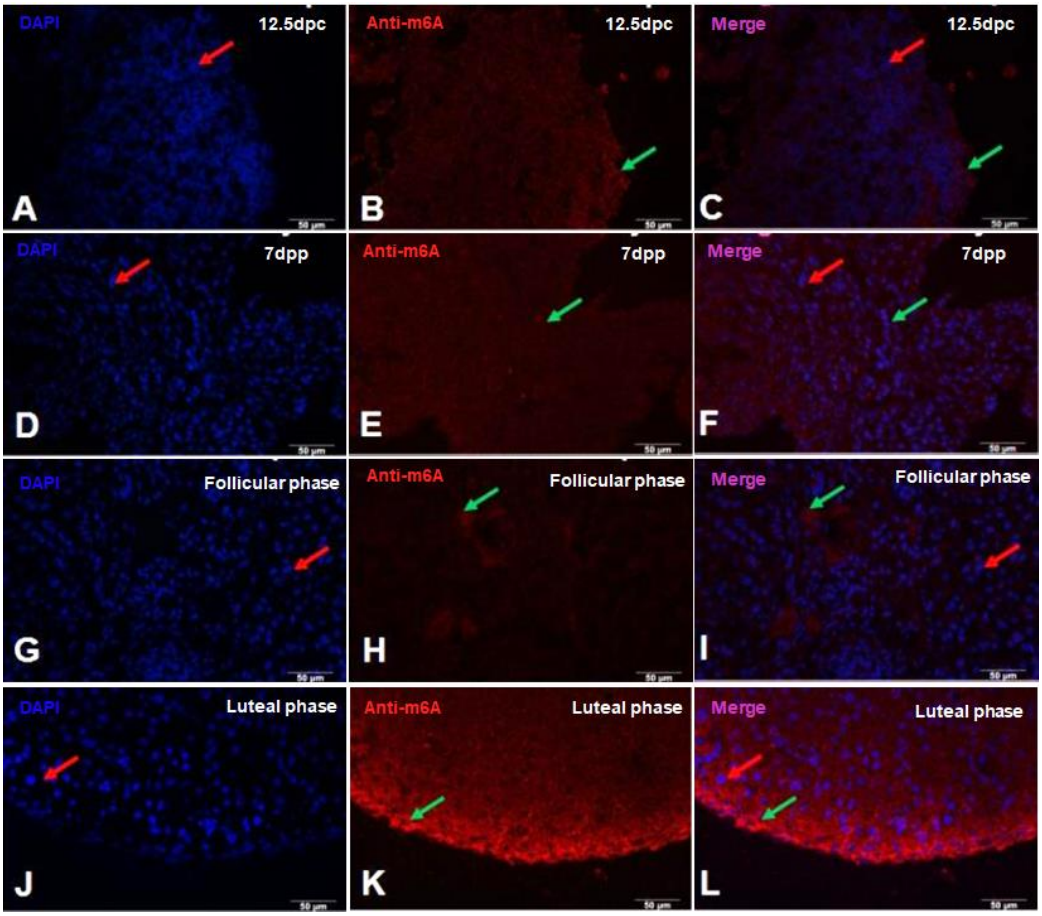

Figure S6: Immunofluorescence patterns of m6A in genital ridge/ovary of female mice at different stages. (A-C) 12.5 dpc. (D-F) 7 dpp. (G-I) Follicular phase. (J-L) Luteal phase. Blue: DNA, Red: m6A. n=12.

Figure S7

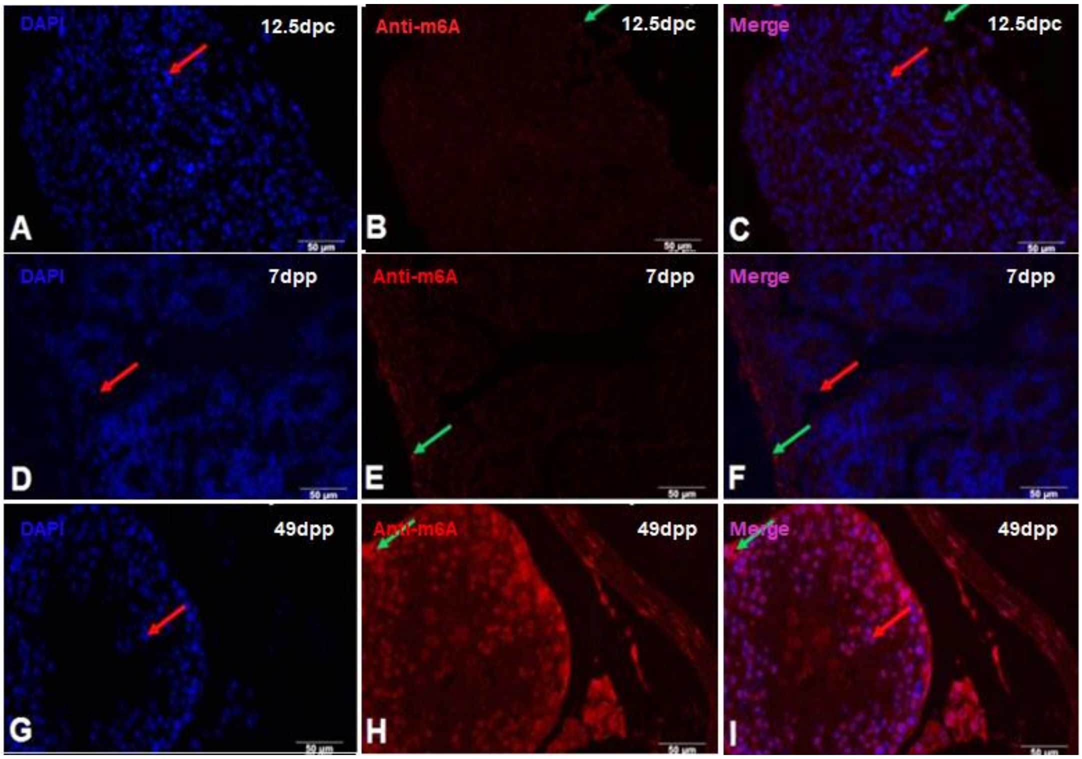

Figure S7: Immunofluorescence patterns of m6A in genital ridge/testis of male mice at different stages.

(A-C) 12.5 dpc. (D-F) 7 dpp. (G-I) Adult stage. Blue: DNA, Red: m6A. n=12.

Figure S8

Compound:A			
Sample Name	Std.Conc	RT	Area
A1	1	1.89	680.318
A2	2	1.90	1673.256
A3	5	1.89	3871.571
A4	100	1.89	8156.543
A5	200	1.91	14405.952
A6	500	1.90	34962.227
A7	1000	1.90	64940.777

Compound:m6A			
Sample Name	Std.Conc	RT	Area
1.1	0.01	3.06	0.857
m6A2	0.02	3.07	1.545
m6A3	0.05	3.05	32.678
m6A4	1	3.07	108.547
m6A5	2	3.08	302.008
m6A6	5	3.06	677.543
m6A7	10	3.06	1462.245

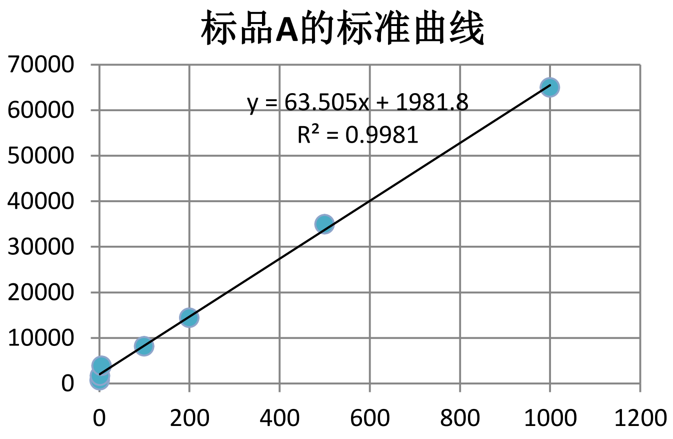

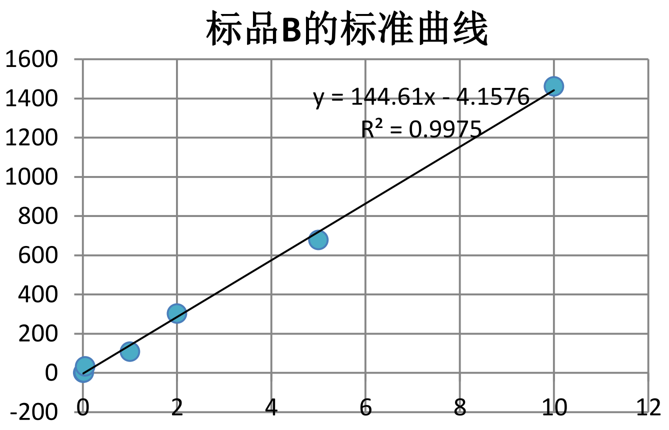

Figure S8: Mass Spectrometry determination and standard curve construction of A and m6A. Std: the Measured Concentration, RT: the Peak Time, Area: the Peak Area, n=6.
